# Development and validation of a machine-learning model for the risk of potentially inappropriate medications in elderly stroke patients

**DOI:** 10.3389/fphar.2025.1565420

**Published:** 2025-05-23

**Authors:** Xiaodan Yang, Qianqian Ye, Mengxiang Zhang, Yuewei Xu, Manqin Yang

**Affiliations:** Department of Pharmacy, The second Affiliated Hospital of Anhui University of Traditional Chinese Medicine, Hefei, China

**Keywords:** stroke, potentially inappropriate medication, machine-learning, elderly patients, prediction model

## Abstract

**Objective:**

To construct a risk prediction model for potentially inappropriate medications (PIM) in elderly stroke patients based on multiple machine-learning algorithms, providing decision support to identify high-risk patients and ensure rational clinical medication use.

**Methods:**

A total of 1,252 discharged stroke patients from a tertiary hospital in Anhui Province, China, were included from January 2023 to December 2024. PIM was assessed using the American Geriatrics Society 2023 Updated Beers Criteria^®^. Univariate analysis identified factors potentially associated with PIM, and the least absolute shrinkage and selection operator regression analysis was applied to select variables. The dataset was randomly split into training and internal validations sets in a 7:3 ratio. Additionally, a dataset independent of the training set in terms of time was selected, consisting of 240 stroke patients diagnosed at the same hospital from January to February 2025, to serve as an external validation cohort. Four machine-learning models, Random Forest, Elastic Net (Enet), Support Vector Machine Classifier, and Extreme Gradient Boosting were built using the meaningful variables identified after selection. The evaluation of machine-learning models was carried out through the discrimination, calibration, and clinical utility. SHapley Additive exPlanation (SHAP) values were utilized to rank the importance of features and to interpret the best-performing model.

**Results:**

Among 1,252 patients, 675 (53.91%) had PIM, with 107 types and 1,140 occurrences of PIM. Both in internal and external validation cohort, Enet performed the best. The area under the curve (AUC) of Receiver Operating Characteristic (ROC) curve of Enet in external validation set was 0.894 (0.854, 0.933). The model’s calibration curve closely followed the ideal curve, and the clinical decision curve showed high net benefit within a threshold probability range of 15%–97%. The results indicate that the Enet prediction model exhibits good accuracy and generalizability, offering a basis for guiding clinical treatment.

**Conclusion:**

The PIM risk prediction model developed using machine-learning can effectively identify PIM, aiding in the implementation of targeted interventions to prevent and reduce the risk of PIM in elderly stroke patients.

## 1 Introduction

Potentially inappropriate medications (PIM) refer to drugs whose effectiveness has not been confirmed or whose risks of adverse drug events outweigh their expected benefits. PIM has a high incidence in clinical practice (Schietzel et al., 2024). Over the past 5 years, the prevalence of PIM in elderly patients has been rising (Suzuki et al., 2022). Elderly patients experience slower metabolism and decreased drug tolerance, which significantly increases the probability of PIM in this population. The presence of PIM in elderly patients can not only reduce medication efficacy but also increase the incidence of adverse drug events, such as falls, fractures, delirium, and even higher rates of disability and mortality (Lockery et al., 2023; Zhou et al., 2025).

Stroke refers to a clinical syndrome characterized by focal or widespread damage to brain tissue caused by sudden cerebrovascular pathological changes, such as ischemic infarction or hemorrhagic lesions. Elderly ischemic stroke patients usually suffer from multiple comorbidities. Studies have shown that 90.0% of middle-aged and elderly stroke patients are comorbid with at least one chronic disease; 13.6% are comorbid with two chronic diseases, 26.9% with three chronic diseases, and 49.4% with four or more chronic diseases (Hu et al., 2024b). Comorbidity increases the complexity of clinical treatment and the number of medications used, which may elevate the risk of PIM in this population. A clinical cross-sectional survey of Chinese stroke patients has confirmed that the incidence of PIM in elderly stroke patients is as high as 69.36% (Wang et al., 2021), far higher than the 33.2% in the general elderly population in China (Zhao et al., 2025). Therefore, it is essential to identify high-risk individuals and populations. Timely and accurate identification and intervention can not only prevent and reduce PIM in elderly patients but also significantly improve the quality of life and healthcare for this population.

Avoiding inappropriate medication use in elderly patients is a real challenge, requiring detailed knowledge of geriatric pharmacotherapy, advanced clinical skills for medication review, and an individualized approach to optimize polypharmacy regimens. Several screening tools are available to assist healthcare providers in selecting medications and reducing the occurrence of PIM in elderly individuals. Among these, the American Geriatrics Society (AGS) Beers Criteria^®^ and STOPP/START are the most widely used standards (O’Mahony et al., 2023; Zhu et al., 2023). In May 2023, the AGS Beers Criteria^®^ was updated to the 2023 version, further enhancing the accuracy and practicality of the standards. The Beers Criteria includes numerous PIMs, and manual evaluation by assessors requires significant time, with high heterogeneity in results due to differences between institutions or assessors. However, current PIM studies largely focus on influencing factors (Chen et al., 2023; Prior et al., 2023). Clinical PIM screening mostly adopts inefficient manual screening methods, which rely entirely on clinical pharmacists for a non-specific approach and incur high labor costs, making it difficult to sustain (Peterson et al., 2014). With the rise of artificial intelligence, machine-learning algorithms have been increasingly applied to develop predictive models (Peng et al., 2022; Li et al., 2022; Guan et al., 2024; Zhang et al., 2024). There is an urgent clinical need for computer algorithms to quickly and accurately identify PIMs to simplify the manual assessment process and reduce heterogeneity.

Machine learning, with its rapid data analysis speed and high accuracy, has shown significant value in prescription drug monitoring, warnings of potentially inappropriate prescribing, and clinical decision support systems (Sharma et al., 2022; Wilhelm et al., 2025). Some researchers have developed PIM prediction models based on GBM, LR, Naive Bayes, neural networks, and RF, but these models have relatively weak predictive power (Best AUC = 0.62) (Chiu et al., 2024). Moreover, these models are based on outpatient data, and the inclusion of candidate features are not comprehensive, missing important patient information such as laboratory test results and weight. Two other studies used LR to construct nomogram models, which showed good performance, but they did not perform internal validation (or, internal verification results are not good.), and the generalizability of the results is questionable (Jiang and Hu, 2023; Ye et al., 2024). Despite preliminary explorations having been conducted on machine models for predicting PIM in the elderly population, there is a lack of research on PIM in elderly stroke patients. To fill this gap, this study applies machine learning to predict PIM in stroke patients. The study first investigates the extent of PIM in elderly patients at a tertiary medical institution in China and the factors leading to these cases, as the first step in identifying strategies that could help reduce drug-related harm in this vulnerable population. Subsequently, the study attempts to develop a risk prediction model using easily accessible patient characteristics, and conducts internal and external validations to ensure the model’s transportability and generalizability. Finally, SHapley Additive exPlanation (SHAP) was employed to enhance interpretability, bridging the gap between complex models and real-world clinical decision-making. This study provides a basis for early identification and intervention in elderly stroke patients with PIM, promoting rational medication use in clinical practice.

## 2 Design

### 2.1 Study design

This single-center, cross-sectional study was conducted from 1 January 2023, to 31 April 2025, at the Second Affiliated Hospital of Anhui University of Chinese Medicine. Founded in 1985, the hospital is currently the largest acupuncture specialty hospital in China, integrating medical care, teaching, research, prevention, healthcare, and rehabilitation. The hospital has 810 approved beds and admits a large number of patients with neurological diseases annually. Since this study is a single-center retrospective study and does not involve human trials, the ethics committee of the Second Affiliated Hospital of Anhui University of Chinese Medicine granted an exemption for this study (Approval No. 2024-zjmc-10). As the patient data was anonymized, informed consent was not required. The study adhered to the principles outlined in the Declaration of Helsinki in all aspects.

### 2.2 Participants

**FIGURE 1:**
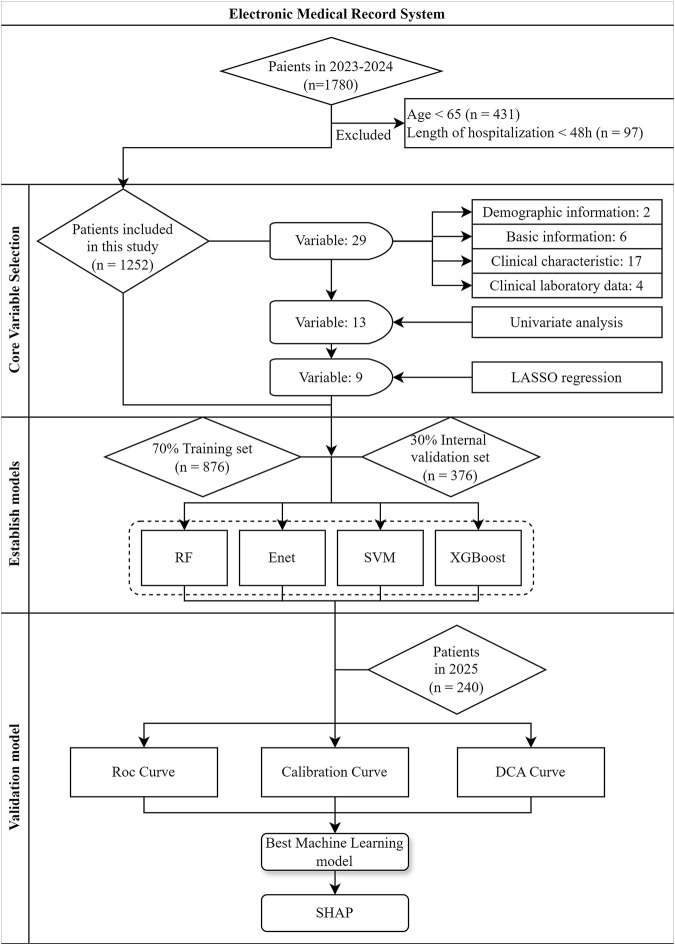
Flowchart for patient recruitment and model establishment

A total of 1,780 discharged patients who met the inclusion criteria between January 2023 and December 2024 were selected using a completely random method. After applying the exclusion criteria, 1,252 patients were finally included in the study. For the external validation group, data independent of the training set in terms of time were selected. Specifically, 240 stroke patients hospitalized at the Second Affiliated Hospital of Anhui University of Chinese Medicine between January and February 2025 were included. The inclusion and diagnostic criteria for these patients were consistent with those of the training set. The process of patient recruitment and model establishment were shown in Figure 1.

#### 2.2.1 Inclusion criteria

• Complete variable information;
• Discharged patients from January 2023 to February 2025
• Discharge diagnosis includes stroke (ischemic stroke, hemorrhagic stroke).

#### 2.2.2 Exclusion criteria

• Age <65 years;
• Hospitalization duration <48 h.

### 2.3 Data collection

A survey form was designed, including the following 29 variables: gender, age, length of hospitalization, clinical disease diagnosis, number of western medicines used, number of discharge diagnosis conditions, attending physician title, albumin, hemoglobin, creatinine, creatinine clearance rate, whether it is the first hospitalization, presence of cognitive impairment, sleep disorders, consciousness disorders, motor disorders, speech disorders, etc.

The initial set of variables was systematically selected based on three key aspects:

• Literature Evidence: A PubMed search strategy ((“predict” OR “risk assessment” OR “diagnosis”) AND (“PIM” OR “potentially inappropriate medication”)) was used to identify relevant studies from 1990 to 2023. Variables significantly associated with PIM were extracted from the literature.
• Clinical Expert Consultation: We consulted three geriatricians and two clinical pharmacists to identify key variables based on clinical experience.
• Data Availability: Variables were prioritized based on structured fields in electronic health records, ensuring that selected variables had clear definitions, standardized documentation, and a missing data rate <10%.

If data were found to be missing or unclear, the research team would contact the patient’s attending physician to collect as much accurate information as possible.

### 2.4 Use of PIMs

The AGS 2023 Updated Beers Criteria^®^ is widely used and applies to all patients aged ≥65 in outpatient, acute, and institutionalized care settings, excluding palliative and hospice care (American Geriatrics Society 2023 updated AGS Beers Criteria^®^ for potentially inappropriate medication use in older adults, 2023). It is one of the most commonly used tools for PIM screening. The AGS 2023 Updated Beers Criteria^®^ not only evaluates PIM medications but also assesses their subcategories. The criteria include five main scales:

• PIMs for elderly patients.
• PIMs with drug-disease or drug-syndrome interactions in elderly patients.
• PIMs that should be used cautiously in elderly patients.
• PIMs that should be avoided in combinations for elderly patients.
• PIMs based on renal function for elderly patients.

The research team reviewed medication information through electronic medical records and conducted PIM evaluations based on the 2023 AGS Updated Beers Criteria^®^. The first author was responsible for the initial identification of PIMs, and the second author verified all PIMs. If there were discrepancies between the two evaluations, the authors discussed and resolved the differences. If a consensus could not be reached, the corresponding author made the final decision. When any medication from the list of criteria was found in the records, it was considered one occurrence of PIM. For drug-drug interaction PIMs, when two or more interacting medications were used together, it was counted as one occurrence of PIM. In all prescriptions, only medications used daily and regularly were included in the analysis. Medications used for short-term illnesses (such as cough medicines for colds, antibiotics for urinary tract infections and pneumonia, etc.), as well as medications used as needed (such as patches or eye drops), were excluded from the analysis.

### 2.5 Data analysis and statistical methods

The study participants were divided into two groups based on the occurrence of PIM: the PIM group and the non-PIM group. Inter-group differences in statistical data were compared, and core variables were selected. The study subjects were randomly split into training and internal validations sets at a 7:3 ratio, and clinical prediction models were constructed and evaluated. Data statistical analysis and graphical plotting were performed using R software (version 4.3.2).

#### 2.5.1 Data preprocessing

Quantitative variables were converted to numeric values, and categorical variables were converted to factor variables. If the percentage of missing data was greater than 20%, those records were excluded from the final dataset. For missing data less than 20%, multiple imputation was performed using the RF regression method in the mice package.

#### 2.5.2 Univariate analysis

For comparing differences between the two groups, categorical variables were analyzed using chi-square tests. For continuous variables with normal distribution and equal variance, independent sample t-tests were applied. For non-normally distributed continuous variables or those with unequal variances, the Mann-Whitney U test was used. The significance level was set at α = 0.05.

#### 2.5.3 Core variable selection

Before constructing the model, the Least Absolute Shrinkage and Selection Operator (LASSO) regression was applied for feature selection to identify the most relevant predictors. To minimize overfitting and identify optimized hyperparameters, 10-fold cross-validation was performed. Variables with P-values <0.05 from inter-group comparisons were selected and further refined using LASSO regression analysis, resulting in a final set of key variables for model inclusion.

#### 2.5.4 Model construction

To achieve the highest prediction performance, four models were constructed: RF(Random Forest), Elastic Net (Enet), Support Vector Machine (SVM), and Extreme Gradient Boosting (XGBoost). These models were selected due to their diversity, extensive use in contemporary clinical prediction, and their demonstrated effectiveness in previous studies (Grazal et al., 2022; Lin et al., 2022). The Enet method combines L1 (Lasso) and L2 (Ridge) regularization, making it particularly well-suited for high-dimensional datasets. It allows for simultaneous variable selection and multicollinearity control (Struck et al., 2019). In clinical prediction models that incorporate multifaceted features such as demographic characteristics, clinical indicators, and medical history, Enet effectively identifies key predictors (e.g., specific comorbidities), thereby improving model interpretability in clinical settings. RF and XGBoost are ensemble learning algorithms that classify data based on the aggregated predictions of multiple decision trees. Their advantages include robustness in handling high-dimensional data, strong predictive capability, and resilience to overfitting. These models are particularly effective in large-scale datasets, offering high accuracy and model stability (Guan et al., 2024). Support Vector Machine (SVM) is a versatile machine-learning algorithm widely used in both classification and regression tasks (Lin et al., 2022). It enhances classification performance by projecting data into higher-dimensional spaces. The optimal hyperparameters were determined using grid search combined with 10-fold cross-validation.

#### 2.5.5 Evaluation of model performance

The evaluation of machine-learning models was conducted based on the discrimination, calibration, and clinical utility in internal validation cohort and external validation cohort. The discrimination of these models was evaluated using the Receiver Operating Characteristic (ROC) curve, with the area under the curve (AUC) being calculated. The AUC with values closer to 1 indicate stronger predictive performance. In clinical prediction models, an AUC greater than 0.8 is generally considered to reflect good discriminatory power. DeLong’s test was used to determine whether differences in AUC values among the models were statistically significant.

Model calibration was evaluated using calibration curves, generated through 1,000 bootstrap resampling iterations. These curves assess the agreement between predicted probabilities and observed outcomes, visualized as scatter plots comparing predicted versus actual event rates. Specifically, all individuals were ranked in ascending order by predicted probabilities and divided into ten equal-sized groups. For each group, the mean predicted probability and the observed event rate were calculated. The scatter plot was then constructed with predicted probability on the x-axis and observed event rate on the y-axis. In a well-calibrated model, the plotted points align closely with the 45° diagonal line, indicating strong concordance between predictions and outcomes. The Brier score, which measures the average squared error between predicted probabilities and actual labels, was used to evaluate model performance; a lower score indicates better model performance.

$$\text{Brier Score}=\frac{1}{N}\sum_{i=1}^{N}{\left(f_{i}-o_{i}\right)}^{2}$$

Among them, “ $f_{i}$ ” denotes the probability predicted by the model, “ $o_{i}$ ”represents the actual result (0 or 1), and *N* stands for the number of samples.

Clinical decision curve analysis (DCA) was used to assess clinical net benefit and validate the model’s clinical applicability. This analysis assesses the usefulness and cost-effectiveness of the predictive model by determining threshold values, evaluating Net Benefits, and establishing decision rules. In the DCA curve, the x-axis represents the threshold probability, while the y-axis represents the net benefit.

$$\text{Net Benefit}=\frac{TP}{n}-\frac{FP}{n}\times \frac{p_{t}}{1-p_{t}}$$

In this context, “n” signifies the sample size, “ $p_{t}$ ” indicates the threshold probability, while “*T*'” and “*FP*” represent the quantities of patients with true positive and false positive results, respectively. Based on this theory, Net Benefit strikes a balance between benefit and loss values. If a subject’s Net Benefit lies within an acceptable range, intervention or appropriate treatment measures will be advised.

There are two extreme scenarios in DCA curve: treating all patients and treating none. The model is considered clinically beneficial only if its curve lies above both extreme cases. Additionally, researchers can determine the optimal threshold probability based on the net benefit, aiding in informed decision-making for clinical interventions.

#### 2.5.6 Model visualization

SHAP algorithm was employed to generate a bee-swarm plot, illustrating the contribution of each feature to the prediction outcomes. Additionally, SHAP force plots were created for selected cases to visualize the impact of individual features on specific samples, providing deeper insights into the model’s decision-making process.

## 3 Results

### 3.1 Patient characteristics and PIMs prescriptions

A total of 1,252 patients were included, with 666 males (53.19%) and 586 females (46.81%), and an average age of 76.61 ± 7.17 years. The three most common diseases among the patients were hypertension (1,001 cases, 79.95%), diabetes (504 cases, 40.26%), and coronary heart disease (240 cases, 19.17%). Among the 1,252 elderly stroke patients, 675 (53.91%) experienced PIM. A total of 107 types of PIM were identified, with 1,140 occurrences.

There were statistically significant differences between the two groups in terms of age, length of hospitalization, number of discharge diagnoses, number of Western medicines used, whether the patient had diabetes, history of falls and fractures, heart failure, atrial fibrillation, sleep disorders, depression, epilepsy, hemoglobin and albumin levels (P < 0.05), as shown in Table 1. The most commonly prescribed PIM drug was spironolactone (12.38%). The most frequent PIM type was “PIMs for elderly patients”, as shown in Tables 2–6.

**TABLE 1:** Univariate analysis of factors related to PIM occurrence.

Variables	Total (N = 1,252)	Non-PIM Group (N = 577)	PIM Group (N = 675)	P-value
Age	76.00 (71.00, 82.00)	75.00 (71.00, 80.00)	76.00 (71.00, 82.00)	0.004
Gender				0.771
Male	666 (53.19)	310 (46.55)	356 (53.45)	
Female	586 (46.81)	267 (45.56)	319 (54.44)	
Weight	63.00 (55.00,70.00)	65.00 (55.00,70.00)	62.00 (55.00,70.00)	0.260
Physician				0.182
Junior	75 (5.99)	33 (44.00)	42 (56.00)	
Intermediate	430 (34.35)	182 (42.33)	248 (57.67)	
Associate Chief	663 (52.96)	318 (47.96)	345 (52.04)	
Chief	84 (6.71)	44 (52.38)	40 (47.62)	
Length of Hospital Stay	15.00 (12.00, 21.00)	15.00 (11.00, 19.00)	15.00 (12.00, 22.00)	<0.001
First Admission				0.089
No	552 (44.09)	239 (43.30)	313 (56.70)	
Yes	700 (55.91)	338 (48.29)	362 (51.71)	
Number of discharged diagnosed	7.00 (5.00, 9.00)	6.00 (5.00, 8.00)	7.00 (6.00, 9.00)	<0.001
Number of Western Medicines Used	10.00 (8.00, 14.00)	9.00 (7.00, 11.00)	12.00 (9.00, 16.00)	<0.001
Hypertension				0.588
No	251 (20.05)	120 (47.81)	131 (52.19)	
Yes	1,001 (79.95)	457 (45.65)	544 (54.35)	
Diabetes				<0.001
No	748 (59.74)	398 (53.21)	350 (46.79)	
Yes	504 (40.26)	179 (35.52)	325 (64.48)	
Hyperlipidemia				1.000
No	1,035 (82.67)	477 (46.09)	558 (53.91)	
Yes	217 (17.33)	100 (46.08)	117 (53.92)	
Coronary Heart Disease				0.379
No	1,012 (80.83)	473 (46.74)	539 (53.26)	
Yes	240 (19.17)	104 (43.33)	136 (56.67)	
Hyperuricemia				0.064
No	1,116 (89.14)	525 (47.04)	591 (52.96)	
Yes	136 (10.86)	52 (38.24)	84 (61.76)	
Parkinson’s Disease				0.942
No	1,207 (96.41)	557 (46.15)	650 (53.85)	
Yes	45 (3.59)	20 (44.44)	25 (55.56)	
History of Falls and Fractures				0.003
No	1,063 (84.90)	509 (47.88)	554 (52.12)	
Yes	189 (15.10)	68 (35.98)	121 (64.02)	
Heart Failure				<0.001
No	1,150 (91.85)	563 (48.96)	587 (51.04)	
Yes	102 (8.15)	14 (13.73)	88 (86.27)	
Atrial Fibrillation				<0.001
No	1,118 (89.30)	558 (49.91)	560 (50.09)	
Yes	134 (10.70)	19 (14.18)	115 (85.82)	
Liver Dysfunction				0.377
No	1,187 (94.81)	551 (46.42)	636 (53.58)	
Yes	65 (5.19)	26 (40.00)	39 (60.00)	
Dementia or Cognitive Impairment				0.191
No	1,048 (83.71)	492 (46.95)	556 (53.05)	
Yes	204 (16.29)	85 (41.67)	119 (58.33)	
Sleep Disorders				<0.001
No	1,160 (92.65)	555 (47.84)	605 (52.16)	
Yes	92 (7.35)	22 (23.91)	70 (76.09)	
Motor Disorders				0.058
No	784 (62.62)	378 (48.21)	406 (51.79)	
Yes	468 (37.38)	199 (42.52)	269 (57.48)	
Consciousness Disorders				0.109
No	1,222 (97.60)	568 (46.48)	654 (53.52)	
Yes	30 (2.40)	9 (30.00)	21 (70.00)	
Aphasia				0.050
No	1,117 (89.22)	526 (47.09)	591 (52.91)	
Yes	135 (10.78)	51 (37.78)	84 (62.22)	
Depression				<0.001
No	1,156 (92.33)	560 (48.44)	596 (51.56)	
Yes	96 (7.67)	17 (17.71)	79 (82.29)	
Epilepsy				<0.001
No	1,214 (96.96)	575 (47.36)	639 (52.64)	
Yes	38 (3.04)	2 (5.26)	36 (94.74)	
Hemoglobin	122.00 (112.00, 133.00)	124.00 (114.00, 133.00)	121.00 (110.00, 133.00)	0.019
Albumin	38.40 (36.00, 41.00)	39.00 (36.50, 41.00)	38.00 (35.00, 40.95)	0.001
Creatinine	63.80 (52.10, 79.50)	64.00 (53.20, 79.00)	63.30 (51.30, 79.80)	0.845
Creatinine Clearance Rate	70.93 (54.82,89.34)	71.43 (58.05, 88.44)	70.23 (51.22, 89.82)	0.168

**TABLE 2:** PIMs for elderly patients.

Organ-system, therapeutic category	Drug(s)	Recommendation	Occurrences	Percentage (%)
Antihistamines	Chlorpheniramine (4), Promethazine (8)	Avoid	12	2.47
Cardiovascular and antithrombotic	Rivaroxaban	Avoid long-term use, consider safer anticoagulants	89	18.31
	Clonidine	Avoid as a first-line antihypertensive	5	1.03
	Nifedipine (Immediate release)	Avoid	5	1.03
	Digoxine	Avoid as first-line treatment for atrial fibrillation or heart failure	21	4.32
	Amiodarone	Avoid using it as the first-line treatment for patients with atrial fibrillation, except in those with heart failure or severe left ventricular hypertrophy	6	1.23
	Warfarin	Avoid using warfarin for non-valvular atrial flutter and venous thromboembolism	4	0.82
Central nervous system	Antidepressant with strong anticholinergic activity: Paroxetine	Avoid	11	2.26
	AntiParkinsonian with strong anticholinergic activity: Benztropine	Avoid	1	0.21
Antipsychotics	Quetiapine (14), risperidone (7), flupentixol and melitracen (35), chlorpromazine (1), tiapride (5), mirtazapine (1)	Avoid, unless used for schizophrenia, bipolar disorder, Parkinson’s disease, or severe depression	63	12.96
Barbiturates	Phenobarbital (1)	Avoid	1	0.21
Benzodiazepines	Clonazepam (24), Diazepam (2), Estazolam (64), Alprazolam (25)	Avoid	115	23.66
Hypnotics that are benzodiazepine receptor agonists (non-benzodiazepie hypnotics)	Right zopiclone (6), Zopiclone (4)	Avoid	10	2.06
Ergoloid mesylates	Dihydroergotoxine mesylate (3)	Avoid	3	0.62
Endocrine	Sulfonylureas: Gliclazide (73), Glimepiride (18), Glipizide (2)	Avoid as first- or second-line treatment, short-acting drugs preferred over long-acting ones	93	19.14
	Insulin (4), Insulin Aspart (2)	Avoid using only short-acting insulin without simultaneous use of basal or long-acting insulin	6	1.23
Gastrointestinal	Metoclopramide	Avoid	12	2.47

**TABLE 3:** PIMs with drug-disease or drug-syndrome interactions in elderly patients.

Disease/Syndrome	Drug(s)	Recommendation	Occurrences	Percentage (%)
Heart failure	NSAIDs and COX-2 inhibitors: Diclofenac (3), Aspirin (8), Ibuprofen (1)	Avoid	12	11.01%
	Cilostazol (2)	Avoid	2	1.83%
	Diltiazem (1)	Avoid or use with caution	1	0.92%
Dementia or cognitive impairment	AntiParkinsonian: Benztropine (1)	Avoid	1	0.92%
	Benzodiazepines: Alprazolam (2), Estazolam (11), Clonazepam (9), Diazepam (1)	Avoid	23	21.10%
	Benzodiazepine receptor agonist: eszopiclone (1)	Avoid	1	0.92%
	Antipsychotics: Tiapride (2), Quetiapine (7), Olanzapine (1)	Avoid	10	9.17%
History of falls or fractures	Antidepressants: Duloxetine hydrochloride, Paroxetine	Avoid	2	1.83%
	Antiepileptics: Sodium valproate (9), Carbamazepine (1), Topiramate (1)	Avoid except for seizures and mood disorders	11	10.09%
	Antipsychotics: Flupentixol and Melitracen (4), Tiapride (2), Risperidone (2), Quetiapine (5)	Avoid	13	11.93%
	Benzodiazepine drugs: Clonazepam (5), Estazolam (19), Alprazolam (3)	Avoid	27	24.77%

**TABLE 4:** PIMs that should be used cautiously in elderly patients.

Drug(s)	Recommendation	Occurrences	Percentage (%)
Dabigatran	Choose Dabigatran with caution for long-term treatment	25	5.47
Ticagrelor	Use with caution, especially for those aged ≥75	28	6.13
Antidepressants: Duloxetine (11), Escitalopram (9), Fluoxetine (4)	Use with caution	24	5.25
Diuretics: Spironolactone (94), Furosemide (82), Hydrochlorothiazide (8), Indapamide (4), Irbesartan and Hydrochlorothiazide (40), Losartan Potassium and Hydrochlorothiazide (10), and Valsartan and Hydrochlorothiazide (1)	Use with caution	239	52.3
Antiepileptics: Sodium valproate (11), Carbamazepine (4)	Use with caution	15	3.28
Tramadol	Use with caution	7	1.53

**TABLE 5:** PIMs based on renal function for elderly patients.

Drug class	Drug(s)	Renal function level	Recommendation	Occurrences	Percentage (%)
Cardiovascular and antithrombotics	Eldoxaban	CrCl15-50 mL/min	Reduce dosage	2	3.03%
	Spironolactone	CrCl<30 mL/min	Avoid	14	21.21%
	Rivaroxaban	CrCl<50 mL/min	Adjust dosage	4	6.06%
	Enoxaparin	CrCl<30 mL/min	Reduce dosage	1	1.52%
CNS and analgesics	Baclofen	eGFR<60 mL.min-1 (1.73m2)-1	Avoid, when avoidance is not feasible, use the lowest dose and monitor for CNS toxicity	2	3.03%
	Levetiracetam	CrCl≤80 mL/min	Reduce dosage	10	15.15%
	NSAIDs: Celecoxib (1), Aspirin (4), Lornoxicam (1)	CrCl<30 mL/min	Avoid	6	9.09%
	Intermediates	CrCl<60 mL/min	Reduce dosage	11	16.67%
Gastrointestinal tract	Famotidine (3), ranitidine (13)	CrCl<50 mL/min	Reduce dosage	16	24.24%

**TABLE 6:** PIMs that should be avoided in combinations for elderly patients.

Object drugs or class	Interacting drug or class	Recommendation	Occurrences	Percentage (%)
RAS inhibitor (ACEI、ARB)	Another RAS inhibitor (ACEI、ARB)	Avoid routine use of ≥ two RAS inhibitors	2	15.38
Antiepileptic drugs; Antidepressants; Antipsychotics; Benzodiazepines; Benzodiazepine receptor agonists hypnotics	These CNS active drugs are used in combination with ≥ three types	Avoid the simultaneous use of ≥ three CNS drugs and reduce the use of CNS drugs as much as possible	10	76.92
Warfarin	Amiodarone	Avoid as much as possible; if simultaneous use is necessary, please closely monitor INR	1	7.69

### 3.2 Clinical variable selection

**FIGURE 2:**
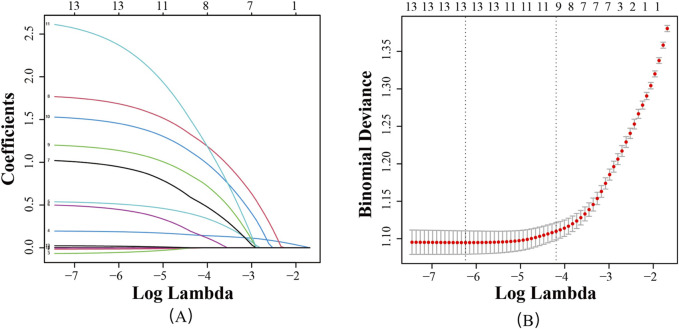
Screening of feature variables using the LASSO regression model. **(A)** Variation characteristics of variable coefficients; **(B)** Tuning parameter(λ) selection in the LASSO model used 10-foldcross-validation.

To mitigate the effects of multicollinearity among variables, 13 variables that showed statistically significant differences in univariate analysis were included in LASSO regression analysis. With the optimal regularization parameter λ set at 0.015, nine potential risk factors for PIM occurrence in stroke patients were identified: epilepsy, atrial fibrillation, sleep disorders, depression, heart failure, diabetes, number of Western medicines used, history of falls and fractures, and length of hospitalization (as shown in Figure 2).

### 3.3 Construction and comparison of four predictive models for PIM

**FIGURE 3:**
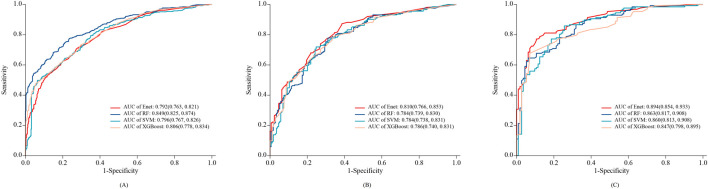
ROC curves of the 4 types of Machine-Learning models in training set **(A)**, internal validation set **(B)** and external validation set **(C)**.

1,252 patients were randomly divided into the training set (876 cases) and the internal validation set (376 cases) from 2023 to 2024. There was no statistically significant difference in PIM incidence between the training and validation sets (53.9% vs. 53.8%, p = 0.975), nor in key baseline characteristics such as the number of medications, presence of heart failure, or atrial fibrillation (p > 0.05), as detailed in Supplementary Table S1. Based on the 9 clinical variables selected by LASSO regression, four machine-learning predictive models were constructed: RF, Enet, SVM, and XGBoost. The results showed that in the internal validation set, the Enet model performed the best among all models, with an ROC-AUC of 0.810 (0.766–0.853), higher than the other models, as shown in Figure 3. DeLong’s test confirmed that the AUC of the Enet model was significantly different from all other models (P < 0.05), whereas the differences among RF, SVM, and XGBoost were not statistically significant (Supplementary Table S2).

The external validation cohort consisted of 240 elderly stroke patients. Among them, 127 (52.91%) experienced PIM. As shown in Figure 3, the Enet model achieved an AUC of 0.894 (0.854–0.933), with a specificity of 0.894 (0.837–0.951) and a sensitivity of 0.772 (0.699–0.845) in the external validation set. The AUC of the other models (RF, SVM, and XGBoost) ranged from 0.847 to 0.863. DeLong’s test indicated that the RF model had a significantly higher AUC than the other models (P < 0.05) (Supplementary Table S3).

**FIGURE 4:**
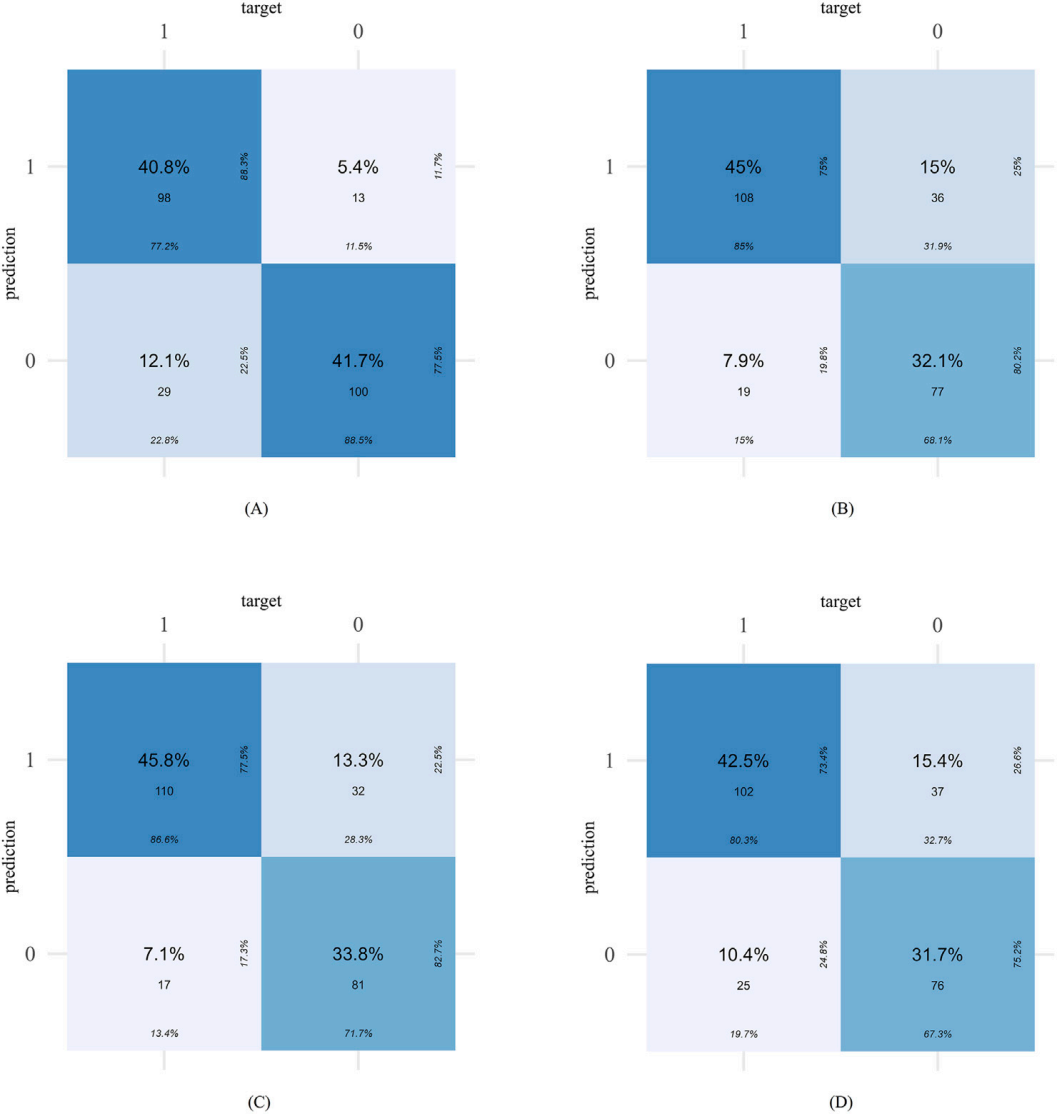
The confusion matrices of different models in external validation set: Enet **(A)**, RF **(B)**, SVM **(C)**, XGboost **(D)**.

The confusion matrices of different models are shown in Figure 4. The Enet model tended to limit the number of false positives, with missed diagnoses of “high-risk PIM patients” being the primary error type. For instance, in the external validation set, the Enet model produced 29 false negatives, considerably more than its 13 false positives. Conversely, the XGBoost model exhibited a more balanced prediction performance, with 55 false negatives and 53 false positives in the internal validation set.

**FIGURE 5:**
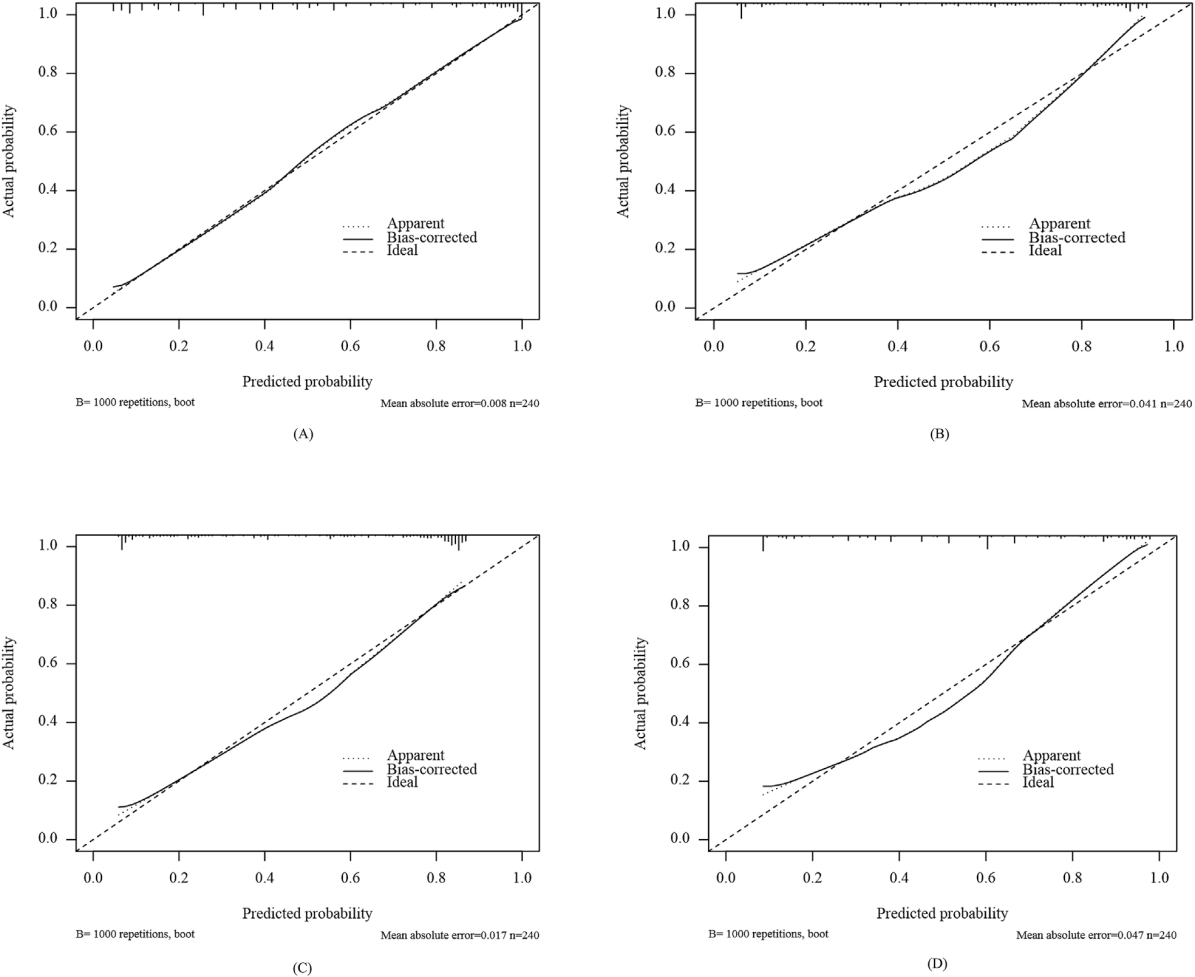
Calibration curves of different models in external validation set: Enet **(A)**, RF **(B)**, SVM **(C)**, XGboost **(D)**.

Calibration curves were plotted to assess the calibration ability of the predictive models. As shown in Figure 5, the calibration curves of the four models in external validation set were plotted: the x-axis represents the predicted PIM risk, and the y-axis represents the actual diagnosed PIM; the diagonal dotted line represents a perfect prediction by an ideal model; the solid line represents the performance of the four machine-learning models, of which a closer fit to the diagonal dotted line represents a better prediction. From the figure, it is evident that the calibration curves of all models are closely approximates the ideal calibration curve. The external validation calibration curve of Enet, as shown in Table 7 achieved a much lower Brier score compared to other models (Enet = 0.130, RF = 0.151, SVM = 0.152, XGBoost = 0.162). The Brier score of 0.130 indicates a good model fit, with high consistency between the actual probability of the outcome and the model’s predicted probability.

**TABLE 7:** Model performance comparison in external validation set.

	Enet	RF	SVM	XGboost
AUC	0.894 (0.854, 0.933)	0.863 (0.817, 0.908)	0.860 (0.813, 0.908)	0.847 (0.798, 0.895)
Accuracy	0.825 (0.771, 0.871)	0.771 (0.712, 0.822)	0.796 (0.739, 0.845)	0.742 (0.681, 0.796)
Precision	0.883 (0.835, 0.931)	0.750 (0.699, 0.801)	0.775 (0.726, 0.823)	0.734 (0.680, 0.787)
F1	0.824 (0.775, 0.872)	0.797 (0.746, 0.848)	0.818 (0.769, 0.867)	0.767 (0.713, 0.820)
Recall	0.772 (0.723, 0.820)	0.850 (0.800, 0.901)	0.866 (0.817, 0.915)	0.803 (0.750, 0.857)
Brier score	0.130 (0.104, 0.156)	0.151 (0.124, 0.177)	0.152 (0.124, 0.180)	0.162 (0.137, 0.188)

Accuracy = (TP + TN)/(TP + TN + FP + FN). Precision = TP/(TP + FP). Recall = TP/(TP + FN). F1 score = 2/([1/Recall] + [1/Precision]). FN, false negatives; FP, false positives; TN, true negatives; TP, true positives.

**FIGURE 6:**
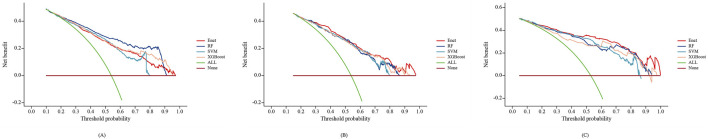
Decision curve analysis of the 4 types of Machine-Learning models in training set **(A)**, internal validation set **(B)** and external validation set **(C)**.

As shown in Figures 6A–C, the DCA curve was used to assess the clinical utility of the PIM model. The results (Figure 6B) indicate that the Enet model provided the most accurate clinical outcome prediction and performed best across the entire threshold range, demonstrating high clinical applicability. When the predicted probability ranged between 15% and 97%, the Enet model showed significant clinical utility in predicting PIM risk among elderly stroke patients.

Based on the combined results of the ROC curve, calibration curve, and DCA curve, the Enet model exhibited superior performance in both internal and external validation, with higher accuracy and better clinical applicability. Therefore, the Enet model is recommended as the preferred predictive model for PIM in elderly stroke patients, followed by the SVM model.

### 3.4 Explainable machine learning

**FIGURE 7:**
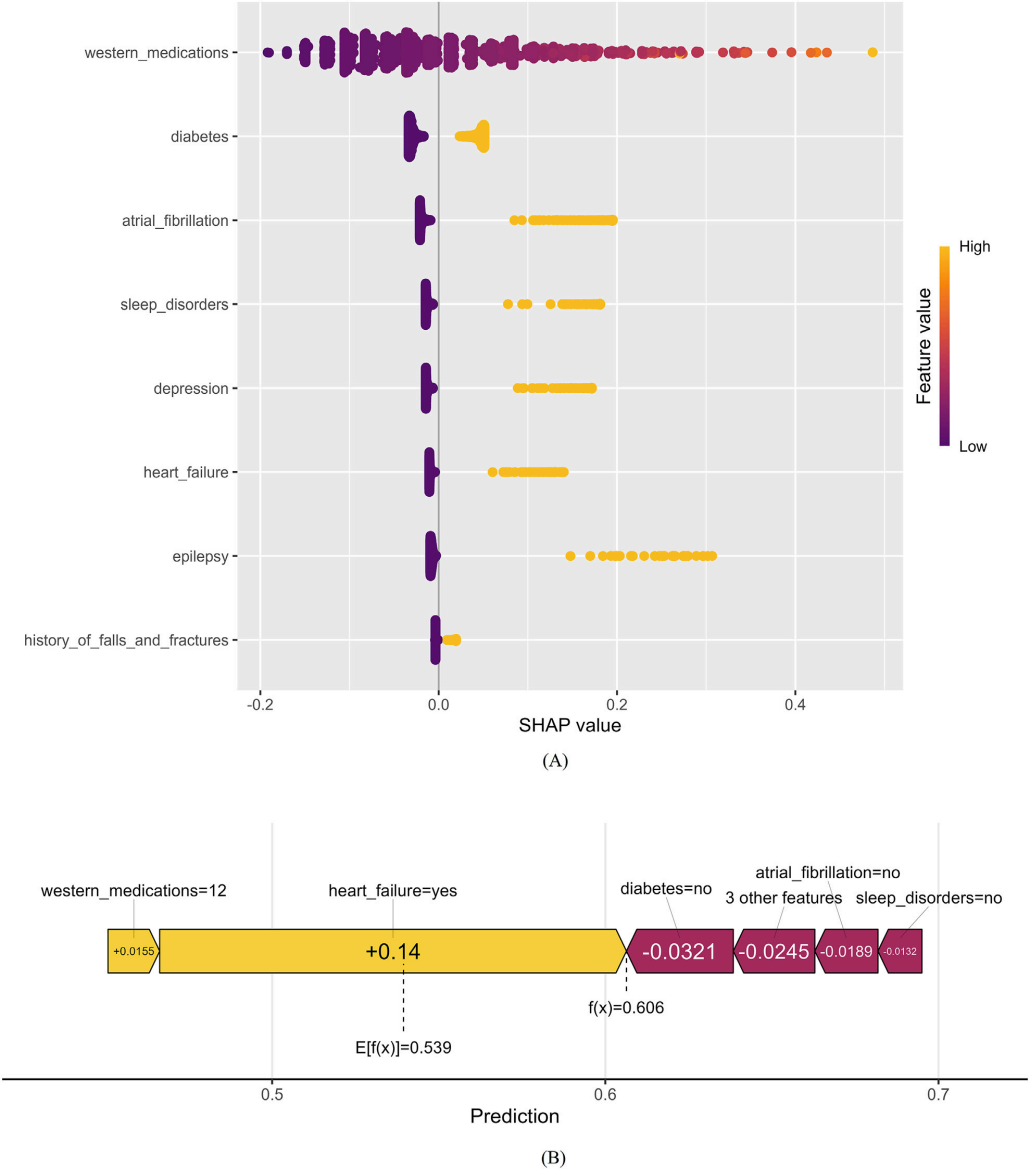
Positive and negative impact explanation of features for predicting PIM using SHAP values. **(A)** Explanation of each feature impact on the PIM in the prediction model by the SHAP values in the Enet. **(B)** Individual efforts by patients with PIM.

Figure 7A presents a comprehensive bee-swarm plot, which visualizes the impact of each feature on Enet’s predictions by incorporating individual feature values. The x-axis represents SHAP values, quantifying the specific influence of each feature on the model’s predictions, while the y-axis lists different features ranked by their contribution to the model’s output. Each data point corresponds to a specific instance, with its position along the x-axis indicating the SHAP value for that feature-instance pair. The eight most important factors in the model were: number of western medications used, diabetes, atrial fibrillation, sleep disorders, depression, heart failure, epilepsy, and history of falls and fractures.

Figure 7B provides an illustrative example of a high PIM-risk individual, demonstrating how the model generates predictions for a specific patient. In this SHAP force plot, the base value of the model is marked as 0.539, while the center-marked value 0.606 represents the final predicted outcome for this sample. The f(x) value represents the actual SHAP value for each feature, obtained by summing the SHAP values of all features with the base value. Variables pulling the prediction toward a higher risk are highlighted with yellow arrows, showing their influence on the final prediction. The SHAP force plot provides an intuitive visualization of individual sample predictions, illustrating how each feature contributes to the prediction step by step.

## 4 Discussion

PIM is highly prevalent in the elderly population and is a major risk factor for adverse drug reactions in older patients, significantly increasing hospitalization and mortality rates, as well as medical costs. The results of this study show a PIM occurrence rate of 53.91% among elderly stroke patients, which is similar to the findings of Matsumoto’s study at Kumamoto Rehabilitation Hospital in Japan (Matsumoto et al., 2022). By using assessment criteria to evaluate patients’ medication patterns, PIM can be identified, improving clinical drug selection and reducing the occurrence of adverse drug reactions. However, manual evaluation is time-consuming, and the differences in the professional levels of healthcare workers at various medical institutions make it unrealistic for most doctors to rely solely on their professional knowledge to assess PIM when prescribing. Therefore, the automation of PIM prediction and screening is of significant importance.

Currently, research on using machine learning to predict PIM remains scarce and has significant limitations as shown in Table 8. Compared to other studies, the variables included in this study were more comprehensive, allowing for evaluation of PIM risk in stroke patients from multiple perspectives. To ensure model validity, both internal validation and temporal external validation were performed. Temporal validation, recommended by the TRIPOD guidelines (Collins et al., 2015), is one of the key external validation strategies. The results provide strong evidence of short-term model robustness (Shen and Wang, 2022; Wu et al., 2023). The optimal model (Enet) achieved a ROC-AUC of 0.894 in external validation, with an overall prediction accuracy of 82.5%, demonstrating strong discriminative ability, high accuracy, and good generalizability. The calibration curve confirmed a high degree of consistency between predicted and actual PIM risk, while DCA analysis indicated substantial clinical net benefit, further supporting the model’s practical applicability. This is the first study to predict PIM in Chinese elderly stroke patients, which use just 8 easily obtainable features with good accuracy.

**TABLE 8:** Comparing of this study with previous studies.

Authors	Sampling population	Range of feature variables	Modeling methods	Internal validation	Model evaluation metrics	AUC
Hu et al. (2024a)	Older adults (>65 years)	15 Variables: basic information, demographic information, medication information	RF, Light-GBM, XGBoost, CatBoost, DF, and TabNet	Yes	Accuracy, precision, recall, F1 scores, subset accuracy and hamming loss	No
Chiu et al. (2024)	Older adults (>65 years)	6 Variables: basic information, demographic information	GBM, LR, naive Bayes, neural networks, and RF	Yes	AUC, negative predicted values, positive predicted values, accuracy, and Brier score	0.62
Jiang and Hu, 2023	Elderly tumor inpatients (>65 years)	19 Variables: basic information, demographic information, clinical characteristics	LR	No	C-index, ROC-AUC, Calibration curve, DCA	0.72
Ye et al. (2024)	Patients with hypertension combined with cerebral infarction (>65 years)	11 Variables: basic information, demographic information, clinical characteristics	LASSO-Logistic regression	Yes	ROC-AUC, Calibration curve	0.85
This study	Elderly patient (>65 years)	29 Variables: basic information, demographic information, clinical characteristics, clinical laboratory data	RF, Enet, SVM, XGBoost	Yes	Accuracy, precision, recall, F1 scores, ROC-AUC, Calibration curve, DCA	0.89

To overcome the “black box” limitation of machine learning models in clinical applications, SHAP analysis was employed to interpret the contribution of each predictor to the final model’s output. SHAP values ranked the eight most important factors influencing PIM risk, in order of contribution: number of western medications used, diabetes depression, atrial fibrillation, sleep disorders, epilepsy, heart failure, history of falls and fractures. Among these, seven out of eight are disease-related characteristics, indicating that underlying comorbidities play a crucial role in PIM risk among elderly stroke patients.

Elderly stroke patients often have underlying conditions such as hypertension, dyslipidemia, and diabetes, and develop various complications post-stroke, including epilepsy, pain, cognitive impairment, sleep disorders, and depression. However, introducing medications to treat these comorbidities and complications blindly may increase the risk of PIM. For example, elderly patients with depression often have limited efficacy from antidepressants, and some studies have even shown that the use of antidepressants may increase the risk of stroke in elderly individuals (Ön et al., 2022). In a study involving 21,805 elderly ischemic stroke patients, 1,835 (8.4%) used antidepressants. Compared with patients who did not use antidepressants, those who used them had a higher incidence of all-cause mortality, all-cause readmission, major adverse cardiac events, depression-related readmissions, and reduced home time (Etherton et al., 2021). In a meta-analysis of 34 randomized controlled trials involving 3,690 elderly patients with severe depression, it was found that the efficacy of antidepressants decreases with age (Calati et al., 2013). The poor efficacy of antidepressants in elderly patients may be due to the burden of comorbid conditions, such as cardiovascular disease and ischemic brain lesions (manifested as white matter hyperintensities in MRI) (Kok and Reynolds, 2017). We suggest using psychological therapy as the first-line treatment for mild to moderate depression in elderly patients (Poletti et al., 2024). Compared to drug treatment, psychological therapy has better tolerability and potential benefits for elderly patients with depression. In a multi-center randomized clinical trial for late-life depression, compared with the control group, psychological therapy (cognitive behavioral therapy, supportive psychotherapy) significantly improved depressive symptoms and reduced sleep disorders and anxiety (Dafsari et al., 2023). In terms of medication, tricyclic antidepressants were most commonly prescribed for PIM in this study, and it is recommended that clinicians consider alternatives such as agomelatine, bupropion, or mirtazapine (Schiavo et al., 2022). This study confirms that the number of Western medications is associated with PIM occurrence. Previous studies have also shown that the risk of PIM increases as the number of medications prescribed increases. Ye et al. (2024) confirmed that in elderly hypertensive patients with ischemic stroke, for each additional medication, the likelihood of PIM increased by approximately 4.12%. In another cross-sectional study, it was found that each additional medication increased the risk of being in a pre-frail or frail state by 8% in patients with blood cancers (Hshieh et al., 2022). In this study, the 1,252 elderly patients had an average of 10 types of Western medicines used during their hospital stay, highlighting the need for clinicians to strengthen prescription reviews for elderly patients receiving multiple medications and improve prescription quality to ensure drug safety. Patients using multiple medications or those requiring drugs with narrow therapeutic ranges need close monitoring to control their drug exposure within the ideal therapeutic window. It is worth noting that the variables selected in our final predictive model, as well as their meanings, appear to be medically consistent and have strong pathophysiological rationale.

This study included 1,252 elderly patient prescriptions, and PIMs were found in 675 patients, with 1,140 occurrences of 107 different types. The most common PIM-related drugs were benzodiazepines, including eszopiclone, alprazolam, and clonazepam, which were most commonly prescribed to elderly stroke patients in the hospital. The high use and prolonged use of benzodiazepines can be attributed to the high insomnia rates in elderly patients. Insomnia is more common in neurological diseases, with insomnia rates in vascular diseases such as stroke ranging from 20% to 37%, in inflammatory diseases ranging from 13.3% to 50%, in epilepsy ranging from 28.9% to 74.4%, and in migraines up to 70% (de Bergeyck and Geoffroy, 2023). Benzodiazepines may be effective for acute insomnia, but their long-term use does not effectively treat sleep disorders (De Crescenzo et al., 2022). Furthermore, many depressed patients are prescribed benzodiazepines, often because depression is misdiagnosed, and benzodiazepines are used to treat insomnia or anxiety, common symptoms of depression. Benzodiazepines are ineffective in treating depression and increase the risk of cognitive impairment, delirium, falls, fractures, and car accidents in the elderly (Liu et al., 2020; Scharner et al., 2022; Carvalho et al., 2024). Moreover, the misuse of benzodiazepines also increases the suicide risk among elderly patients (Schepis et al., 2019). Before using such psychoactive medications, the risks and benefits should be clearly assessed. For elderly patients, it is recommended that these medications not be used for more than 4 weeks, regardless of indications. Additionally, lower doses should be used for elderly populations (Kummer et al., 2024). Aside from medication, cognitive behavioral therapy is considered the first-line treatment for chronic insomnia in elderly patients. Several clinical studies have confirmed the efficacy of cognitive behavioral therapy for elderly patients with sleep disorders (Furukawa et al., 2024). Other studies have shown that behavioral therapy is a feasible and acceptable post-stroke intervention, significantly improving fatigue and depressive symptoms in the elderly (Herron et al., 2018; Nguyen et al., 2019).

Diuretics were another common PIM in this study. Diuretics are commonly used in elderly cardiovascular patients, particularly those with hypertension, for their ability to reduce edema and maintain blood flow stability. However, elderly patients are more sensitive to diuretics, which can lead to hypokalemia and hyponatremia, especially with hydrochlorothiazide (Krogager et al., 2020). Diabetes, a common chronic disease, was present in 40.26% of the elderly stroke patients in this study. Due to the significant risk of hypoglycemia and cardiovascular events in elderly patients using sulfonylureas, this class of drugs is classified as inappropriate for use in the elderly by the AGS Beers Criteria^®^. This study found 93 occurrences of such PIMs, with long-acting sulfonylureas such as gliclazide and glimepiride being the most common. It should be noted that the guidelines suggest that if sulfonylureas must be used, short-acting sulfonylureas like glipizide should be considered.

The core innovation of this study lies in establishing a pre-screening accelerator for clinical decision-making rather than replacing existing assessment systems. AI-based clinical algorithms should enhance, rather than replace, human intelligence. In complex and uncertain scenarios, AI tools can provide reliable, reproducible decision support or assist clinicians when uncertainties arise.

This study has three key strengths. First, no prior research has developed a PIM risk prediction model specifically for elderly stroke patients. By leveraging machine learning, this study offers a valuable reference for future clinical research. Second, the final Enet model includes only eight easily accessible variables, ensuring high clinical practicality. Third, the use of SHAP enhances model interpretability, bridging the gap between complex machine-learning algorithms and real-world clinical decision-making, thereby improving trust and usability among healthcare providers.

However, this study has certain limitations. First, the retrospective single-center design inherently risks selection bias, despite rigorous methodological controls. Although internal and temporal external validation support model robustness, geographic variations in prescribing practices may necessitate localized recalibration prior to cross-institutional implementation. Additionally, as a data-driven model, it primarily identifies complex variable associations and should be used for risk stratification rather than direct clinical decision-making. The temporal sequence between predictive variables and outcomes remains uncertain, necessitating prospective studies for further causal exploration. Second, although external validation showed good results, it is impossible to predict future changes in prescribing patterns due to the rapid updates in medications and prescribing habits in healthcare institutions. The establishment of this model is highly dependent on the Beers criteria (2023 version). As a result, if the Beers Criteria is updated, the model would require recalibration. Third, considering the moderate sample size of this study (n = 1,252 + 240) and the need for clinical interpretability, deep learning methods (such as deep neural networks) were not included in this study, despite their outstanding performance in handling imbalanced data and their excellent discriminative and calibration abilities. Future studies with larger cohorts could explore ensemble learning techniques to enhance predictive performance further. Overall, this model provides a foundation for future research, and prospective multi-center studies are necessary to validate its effectiveness further.

## 5 Conclusion

The principle of “treating disease before it develops” is a significant advantage in Chinese medicine’s approach to health and disease. Early identification of PIM in elderly patients is highly beneficial. This study actively explored the risk factors associated with PIM occurrence in elderly stroke patients and developed a simple and understandable PIM risk warning model. This model can quickly and accurately identify PIM at the initiation of medication, simplifying the manual assessment process and reducing the heterogeneity between different institutions and evaluators. It ensures rational clinical medication use and reduces PIM occurrence in elderly patients. The intention behind developing this model is to provide a convenient and practical tool for rational drug use in the elderly, and healthcare workers should increase their awareness of PIM risk, considering individual patient circumstances when making decisions and balancing the benefits and risks of medication therapy. However, the risk prediction model requires prospective and multi-center studies for further validation of its effectiveness.

## Data Availability

The raw data supporting the conclusions of this article will be made available by the authors, without undue reservation.
