# Supplementary material for: Development and validation of a machine-learning model for the risk of potentially inappropriate medications in elderly stroke patients
Source: Front Pharmacol. 2025 May 23;16:1565420. doi: 10.3389/fphar.2025.1565420 (PMC12141006; doi:10.3389/fphar.2025.1565420)
Supplement: Supplementary file 1 [file Supplementaryfile1.doc]

**Supplementary Table1. Comparison of clinical data between training set and validation set**

| Variables | Total (n = 1252) | Training (n = 875) | Validation (n= 377) | p |
| --- | --- | --- | --- | --- |
| Age | 76.00 (71.00, 82.00) | 76.00 (71.00, 82.00) | 76.00 (70.00, 81.00) | 0.257 |
| Gender |  |  |  | 0.352 |
| Male | 666 (53.2) | 473 (54.1) | 193 (51.2) |  |
| Female | 586 (46.8) | 402 (45.9) | 184 (48.8) |  |
| Weight | 63.00 (55.00, 70.00) | 64.00 (55.00, 70.00) | 62.00 (55.00, 70.00) | 0.080 |
| Physician |  |  |  | 0.098 |
| Junior | 663 (53.0) | 481 (55.0) | 182 (48.3) |  |
| Intermediate | 75 (6.0) | 50 (5.7) | 25 (6.6) |  |
| Associate Chief | 84 (6.7) | 61 (7.0) | 23 (6.1) |  |
| Chief | 430 (34.3) | 283 (32.3) | 147 (39.0) |  |
| Length of Hospital Stay | 15.00 (12.00, 21.00) | 15.00 (12.00, 20.00) | 15.00 (11.00, 21.00) | 0.504 |
| First Admission |  |  |  | 0.130 |
| No | 552 (44.1) | 398 (45.5) | 154 (40.8) |  |
| Yes | 700 (55.9) | 477 (54.5) | 223 (59.2) |  |
| Number of discharged diagnosed | 7.00 (5.00, 9.00) | 7.00 (5.00, 9.00) | 7.00 (5.00, 9.00) | 0.912 |
| Number of Western Medicines Used | 10.00 (8.00, 14.00) | 10.00 (8.00, 14.00) | 10.00 (7.00, 13.00) | 0.245 |
| Hypertension |  |  |  | 0.599 |
| No | 251 (20.0) | 172 (19.7) | 79 (21.0) |  |
| Yes | 1001 (80.0) | 703 (80.3) | 298 (79.0) |  |
| Diabetes |  |  |  | 0.779 |
| No | 748 (59.7) | 525 (60.0) | 223 (59.2) |  |
| Yes | 504 (40.3) | 350 (40.0) | 154 (40.8) |  |
| Hyperlipidemia |  |  |  | 0.552 |
| No | 1035 (82.7) | 727 (83.1) | 308 (81.7) |  |
| Yes | 217 (17.3) | 148 (16.9) | 69 (18.3) |  |
| Coronary Heart Disease |  |  |  | 0.966 |
| No | 1012 (80.8) | 707 (80.8) | 305 (80.9) |  |
| Yes | 240 (19.2) | 168 (19.2) | 72 (19.1) |  |
| Hyperuricemia |  |  |  | 0.434 |
| No | 1116 (89.1) | 776 (88.7) | 340 (90.2) |  |
| Yes | 136 (10.9) | 99 (11.3) | 37 (9.8) |  |
| Parkinson’s Disease |  |  |  | 0.855 |
| No | 1207 (96.4) | 843 (96.3) | 364 (96.6) |  |
| Yes | 45 (3.6) | 32 (3.7) | 13 (3.4) |  |
| History of Falls and Fractures |  |  |  | 0.125 |
| No | 1063 (84.9) | 734 (83.9) | 329 (87.3) |  |
| Yes | 189 (15.1) | 141 (16.1) | 48 (12.7) |  |
| Heart Failure |  |  |  | 0.459 |
| No | 1150 (91.9) | 807 (92.2) | 343 (91.0) |  |
| Yes | 102 (8.1) | 68 (7.8) | 34 (9.0) |  |
| Atrial Fibrillation |  |  |  | 0.467 |
| No | 1118 (89.3) | 785 (89.7) | 333 (88.3) |  |
| Yes | 134 (10.7) | 90 (10.3) | 44 (11.7) |  |
| Liver Dysfunction |  |  |  | 0.500 |
| No | 1187 (94.8) | 832 (95.1) | 355 (94.2) |  |
| Yes | 65 (5.2) | 43 (4.9) | 22 (5.8) |  |
| Dementia or Cognitive Impairment |  |  |  | 0.160 |
| No | 1048 (83.7) | 724 (82.7) | 324 (85.9) |  |
| Yes | 204 (16.3) | 151 (17.3) | 53 (14.1) |  |
| Sleep Disorders |  |  |  | 0.868 |
| No | 1160 (92.7) | 810 (92.6) | 350 (92.8) |  |
| Yes | 92 (7.3) | 65 (7.4) | 27 (7.2) |  |
| Motor Disorders |  |  |  | 0.807 |
| No | 784 (62.6) | 546 (62.4) | 238 (63.1) |  |
| Yes | 468 (37.4) | 329 (37.6) | 139 (36.9) |  |
| Consciousness Disorders |  |  |  | 0.110 |
| No | 1222 (97.6) | 858 (98.1) | 364 (96.6) |  |
| Yes | 30 (2.4) | 17 (1.9) | 13 (3.4) |  |
| Aphasia |  |  |  | 0.002 |
| No | 1117 (89.2) | 796 (91.0) | 321 (85.1) |  |
| Yes | 135 (10.8) | 79 (9.0) | 56 (14.9) |  |
| Depression |  |  |  | 0.659 |
| No | 1156 (92.3) | 806 (92.1) | 350 (92.8) |  |
| Yes | 96 (7.7) | 69 (7.9) | 27 (7.2) |  |
| Epilepsy |  |  |  | 0.604 |
| No | 1214 (97.0) | 847 (96.8) | 367 (97.3) |  |
| Yes | 38 (3.0) | 28 (3.2) | 10 (2.7) |  |
| Hemoglobin | 122.00 (112.00, 133.00) | 123.00 (112.00, 133.00) | 122.00 (111.00, 133.00) | 0.850 |
| Albumin | 38.40 (36.00, 41.00) | 38.40 (36.00, 41.00) | 38.60 (36.00, 41.00) | 0.978 |
| Creatinine | 63.80 (52.10, 79.50) | 64.00 (52.50, 78.90) | 62.10 (50.50, 80.00) | 0.387 |
| Creatinine Clearance Rate | 70.93 (54.82, 89.34) | 72.41 (54.97, 88.56) | 69.62 (54.83, 93.71) | 0.751 |
| PIM, n (%) |  |  |  | 0.975 |
| no | 577 (46.1) | 403 (46.1) | 174 (46.2) |  |
| yes | 675 (53.9) | 472 (53.9) | 203 (53.8) |  |

**Supplementary Table 2. DeLong's AUC Comparison Results in internal validation set**

| **Contrast** | **z** | **p. value** |
| --- | --- | --- |
| Enet vs. RF | 2.850 | 0.004 |
| Enet vs. SVM | 2.025 | 0.043 |
| Enet vs. XGBoost | 2.155 | 0.031 |
| RF vs. SVM | -0.269 | 0.788 |
| RF vs. XGBoost | -0.495 | 0.621 |
| SVM vs. XGBoost | -0.154 | 0.878 |

**Supplementary Table 3. DeLong's AUC Comparison Results in external validation set**

| **Contrast** | **z** | **p. value** |
| --- | --- | --- |
| Enet vs. RF | 3.009 | 0.003 |
| Enet vs. SVM | 3.808 | <0.001 |
| Enet vs. XGBoost | 4.086 | <0.001 |
| RF vs. SVM | 2.122 | 0.034 |
| RF vs. XGBoost | 2.576 | 0.010 |
| SVM vs. XGBoost | -0.017 | 0.987 |
